# Computational Study of Quinolone Derivatives to Improve their Therapeutic Index as Anti-*malaria* Agents: QSAR and QSTR

**Published:** 2015

**Authors:** Maryam Iman, Asghar Davood, Ali Khamesipour

**Affiliations:** a *Chemical Injuries Research Center, Baqiyatallah University of Medical Sciences, Tehran, Iran. *; b *Department of Medicinal Chemistry, Faculty of Pharmacy, Pharmaceutical Sciences Branch, Islamic Azad University, Tehran, Iran (IAUPS).*; c *Center for Research and Training in Skin Diseases and Leprosy, Tehran University of Medical Sciences, Tehran, Iran.*

**Keywords:** Cytotoxicity, Quinolone, QSAR, QSTR, Malaria

## Abstract

Malaria is a parasitic disease caused by five different species of *Plasmodium*. More than 40% of the world’s population is at risk and malaria annual incidence is estimated to be more than two hundred million, malaria is one of the most important public health problems especially in children of the poorest parts of the world, annual mortality is about 1 million. The epidemiological status of the disease justifies to search for control measures, new therapeutic options and development of an effective vaccine. Chemotherapy options in malaria are limited, moreover, drug resistant rate is high. In spite of global efforts to develop an effective vaccine yet there is no vaccine available. In the current study, a series of quinolone derivatives were subjected to quantitative structure activity relationship (QSAR) and quantitative structure toxicity relationship (QSTR) analyses to identify the ideal physicochemical characteristics of potential anti-*malaria* activity and less cytotoxicity. Quinolone with desirable properties was built using HyperChem program, and conformational studies were performed through the semi-empirical method followed by the PM3 force field. Multi linear regression (MLR) was used as a chemo metric tool for quantitative structure activity relationship modeling and the developed models were shown to be statistically significant according to the validation parameters. The obtained QSAR model reveals that the descriptors PJI2, Mv, PCR, nBM, and VAR mainly affect the anti-*malaria* activity and descriptors MSD, MAXDP, and X1sol affect the cytotoxicity of the series of ligands.

## Introduction

Malaria is a parasitic disease which is caused in human mainly by five species of *Plasmodium*, called *P. falciparum,P. vivax, P. malariae, P. ovale* spp., and *P. knowlesi *(1). Almost all species of human malaria parasites have a similar life cycle consists of sexual phase of multiplications, which occurs in specific species of Anopheline mosquitoes and an asexual phase of multiplications (schizogony) which take places in blood cells of the vertebrate host which might follow with invasion to the liver cells (2).

Malaria estimated annual incidence rate is about 219 million people worldwide and still considered as one of the most important public health problems especially in children of the poorest parts of the world. Currently, more than 40% of the world’s population are at risk of malaria and the annual mortality is about 1 million (3). Although more than 50 years ago the worldwide malaria eradication program was endorsed but yet high mortality and morbidity caused by malaria especially *P. falciparum* (WHO: World Malaria Report, 2013), studies in different aspects of the disease are highly encouraged. Every available approach should be used to design safe and effective anti-*malaria* drugs (4-6).

Regardless of global efforts to develop an effective vaccine against malaria and valuable publications yet no vaccine is available against malaria (7, 8). 

Current epidemiological and treatment situations of malaria urge to use different approaches to develop new tools for malaria treatment (9). The rate of drug resistance in malaria is high (3), drug resistance in malaria defined as when the parasite survive or multiply, regardless of administration of enough dose of the drug which is absorbed (WHO technical report series, 1965). The speed of development of new anti-*malaria* drugs should be faster than rapid spread of drug resistance. In drug development it should be considered that each stage of malaria life cycle shows susceptibility to a specific anti-*malaria* drug so development of several drugs might be needed to control the disease (10). 

Quantitative Structure Activity Relationship (QSAR) and Quantitative Structure Toxicity Relationship (QSTR) models are mathematical equations relating the chemical structure of a substance to the ‎biological activity and toxicity, respectively (11-21). The current QSAR models are developed based on the resistant malarial strains TM90-C2B (chloroquine, mefloquine, pyrimethamine, and atovaquone resistant) and for cytotoxicity against J774 mammalian of a set of ‎‎28, quinolone derivatives which have already synthesized (22). To select the set of descriptors that are more relevant to EC_50_ of the desired compounds, MLR models were built and QSAR and QSTR equations with stepwise selection and ‎elimination of variables were established using SPSS and Matlab software.‎

## Experimental

*QSAR and QSTR equations*

The biological data used in this study were anti-*malaria* activities (EC_50_), and cytotoxicity of 28 quinolone derivatives against multidrug resistant *p. falciparum *(TM90-C2B) and J774 mammalian, respectively (21). 

As reported previously structures of desired compounds 1 to 28 (Table 1) were built and optimized using HyperChem software (version 7, Hypercube Inc.) (11-15). A large number of molecular descriptors were calculated using HyperChem (Table 2) and Dragon software (23, 24). Dragon software was used to calculate different functional groups, topological, geometrical, and constitutional descriptors for each molecule. The calculated descriptors were collected in a data matrix wherein the number of rows and columns were the number of molecules and descriptors, respectively. Some of the chemical parameters including molecular volume (V), molecular surface area (SA approx), surface area (SA grid) hydrophobicity (LogP), hydration energy (HE), refractivity (Rf), molecular polarizability (MP) and different quantum chemical descriptors including dipole moment (DM) and the highest occupied molecular orbital (HOMO) energies are shown in Table 2. 

Briefly, after removing the constant or near-constant descriptors to decrease the redundancy existed in the descriptor data matrix, the correlation of descriptors with each other and with the activity and cytotoxicity (pEC_50_) of the molecules were examined and collinear descriptors (*i.e.* r>0.9) were detected. Among the collinear descriptors, the one which shows the highest correlation with activity or cytotoxicity was retained and the others were removed from the data matrix. To select the set of descriptors that were most relevant to the anti-*malaria* activities (pEC_50_) and cytotoxicity, MLR models were built and the QSAR equations with stepwise selection and elimination of variables were established using MLR method (25). SPSS (version 18) and Matlab (version 7.6.0, R2008a) software were used for MLR regression method (11-16). 

 In the case of each regression problem, SPSS produced many models and were ranked based on standard error of calibration and coefficient of multiple determinations, wherein some models had a large number of input variables and thus they were over-fitted. To hinder obtaining over-fitted models, the generated QSAR and QSTR models were validated by the leave-one out cross-validation procedure to check the predictability and robustness. A balance between the high cross-validation correlation coefficient and low number of descriptors were used as the criterion for model selection. The overall prediction abilities of the final models were assessed by using a prediction set containing about 25% of the original molecules. To do so, the data set of activity was randomly classified to calibrate and predict the sets. The model coefficients were calculated using calibration data and then were used to calculate the anti-*malaria* activities of the molecules in the prediction set. 

**Table 1:** The chemical structure of quinolone derivatives.

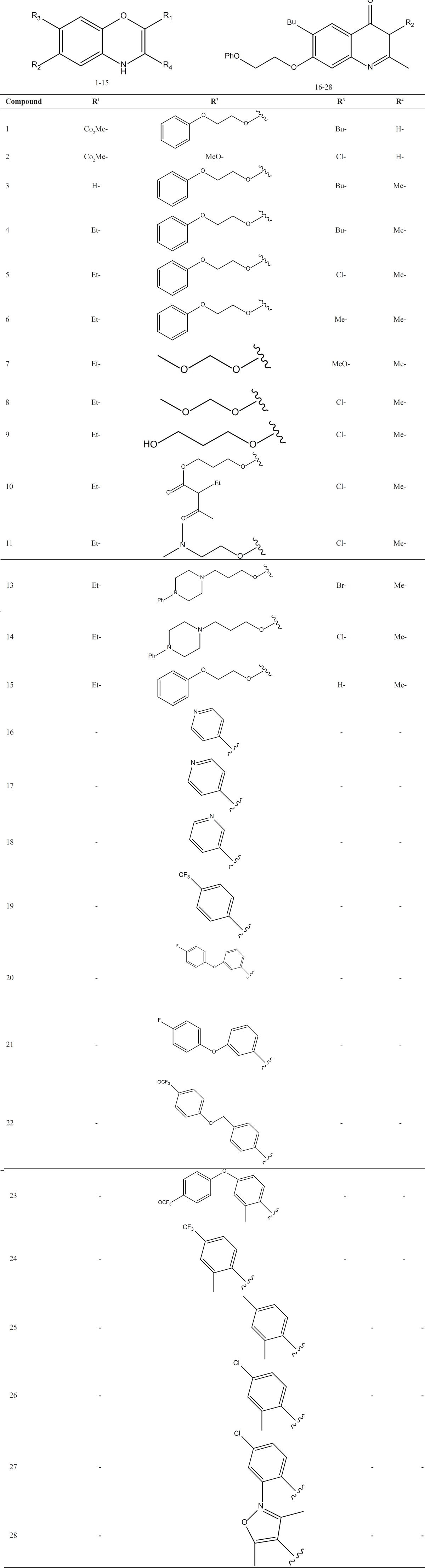

**Table 2:** Calculated properties of quinolones using the HyperChem software.

**Compound**	**surface area(approx)**	**Surface area(grid)**	**volume**	**hydration energy**	**log p**	**refractivity**	**polarizability**	**dipolemoment**	**homo** a
1	637.07	685.75	1152.78	-5.72	3.75	109.05	42.11	5.62	-8.78
2	396.06	426.22	680.98	-4.82	1	64.46	24.52	5.99	-8.77
3	604.95	661	1104.66	-5.19	3.73	105.21	40.19	4.83	-8.59
4	655.37	701.42	1195.12	-4.02	4.4	114.17	43.86	4.54	-8.49
5	558.7	617.37	1030.75	-6.36	3.26	100.13	38.45	5.12	-8.53
6	559.69	618.08	1038.63	-5.65	3.21	100.37	38.35	4.50	-8.46
7	567.72	625.52	1058.52	-7.67	2.49	101.79	38.99	5.43	-8.32
8	467.8	492.18	804.34	-3.95	1.85	75.43	28.79	6.72	-8.49
9	501.99	522.28	860.37	-8.9	1.25	80.74	30.62	7.21	-8.50
10	685.09	701.64	1184.64	-3.18	2.75	108.96	41.8	4.75	-8.53
11	526.28	586.18	996.12	-3.42	1.28	96.67	36.7	6.69	-8.49
12	566.98	559.29	925.33	-1.81	1.62	87.6	33.17	6.1	-8.47
13	565.79	623.43	1047.37	-6.24	3.54	102.95	39.14	5.13	-8.66
14	622.7	724.29	1261.99	-2.88	3.63	129.01	48.91	6.65	-8.48
15	528.2	595.95	989.94	-7.03	2.74	95.33	36.52	4.65	-8.56
16	668.81	760.73	1303.62	-6.27	5.22	129.66	49.85	4.63	-8.47
17	656.24	754.64	1289.86	-7.81	4.84	127.44	49.14	6.38	-8.66
18	656.41	753.56	1292.28	-7.71	4.84	127.44	49.14	6.00	-8.6
19	731.13	804.68	1381.12	-5.7	6.1	135.64	51.41	7.57	-8.72
20	745.17	826.25	1407.73	-7.76	6.9	137.24	52.05	6.54	-8.61
21	782.61	885.75	1550.89	-9.31	6.79	156.12	60.06	5.84	-8.55
22	885.15	988.68	1707.67	-10.05	8.43	168.31	64.18	5.87	-8.56
23	864.51	974.07	1697.37	-10.26	8.8	168.52	64.18	5.51	-8.59
24	748.7	828.23	1430.31	-5.03	6.57	140.68	53.25	7.41	-8.79
25	731.04	814.43	1400.7	-4.35	6.16	139.75	53.52	4.39	-8.55
26	735.48	815.64	1393.25	-5.51	6.24	135.85	51.32	7.15	-8.78
27	724.51	808.52	1391.1	-5.15	6.21	139.51	53.61	5.39	-8.64
28	683.76	773.38	1333.12	-9.05	3.83	131.6	49.97	6.73	-8.67

a highest occupied molecular orbital.

## Results and Discussion

*QSAR equation*

Based on the procedure explained in the Materials and Methods section, using a stepwise multiple linear regression method, the following five-parametric equation 1 (Equation 1) was derived for quinolones 1 to 28.

In the QSAR and QSTR equations, n is the number of data points, R2 is the correlation coefficient, S is the standard deviation, F is the Fisher’s F-value and q2 is the leave-one-out (LOO) cross validated coefficient that was obtained by a multiple linear regression. 

The anti-*malaria* activities of the quinolone derivatives were tested against multidrug resistant *P. falciparum* (TM90-C2B). Herewith, we explain QSAR model for anti-*malaria* activity against TM90-C2B and cytotoxicity against the J774 mammalian in equations 1 and 2, respectively.

Equation (1): 

pEC_50_= (-18.1 ± 9.02) – (8.8 ± 1.7) PJI2 – (12.99 ± 8.18) Mv + (29.6 ± 3.58) PCR – (0.68 ± 0.11) nBM + (0.03 ± 0.006) VAR

 n=28, F=33.157, R^2^=0.92, S=0.89, p<0.000, q^2^=0.71

Equation 1 explains 92% of the variance in pEC_50_ data wherein the relative error prediction (REP) of the equation is shown in Table 5. This equation describes the effect of PJI2, Mv, PCR, nBM and VAR indices on the anti TM90-c2B activity. The PJI2 and VAR are among topological descriptors and Mv and nBM are among constitutional descriptors. PCR is among walk and path counts descriptor that show ratio of multiple path count over path count.

**Figure 1:**
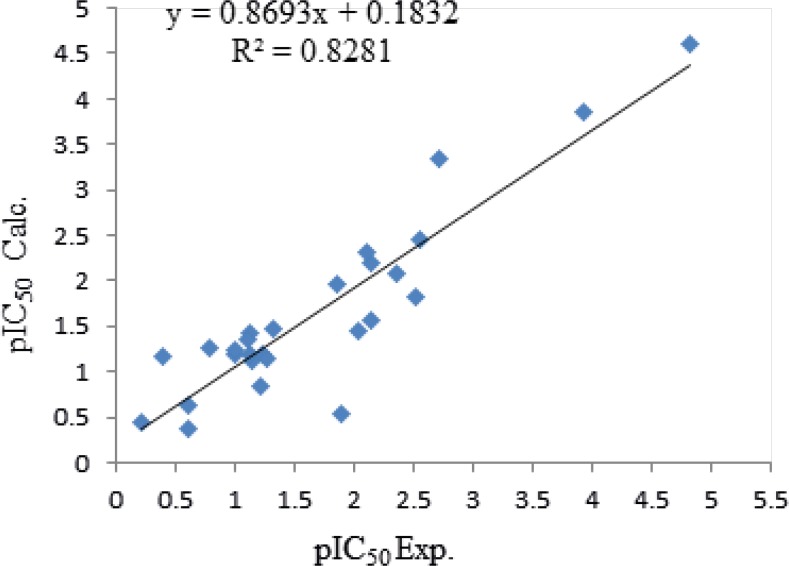
Plot of cross-validated calculated activity of TM90-C2B obtained by QSAR equation 1.

Equation 1 indicates that PCR and VAR demonstrate positive contribution and PJI2, Mv, and nBM show a negative contribution towards the anti-*malaria *activities. Comparison of the coefficient of descriptors reveals that anti-*malaria* (TM90-c2B) activities might be affected mainly by PCR, Mv, and PJI2 with 29.6, 12.99 and 8.8 values, respectively. The calculated pEC_50_ using equation 1 is presented in Table 3 and the graphical representation of cross validated calculated activity and the experimental values using equation 1 are presented in Figure 1. The correlation coefficient matrix for the descriptors that were used in the MLR equation 1 is shown in Table 4. Based on the equation 1, in order to design a new ligand with high potency, substitutions with high amount of PCR and low amounts of Mv and PJI2 should be considered.

**Table 3:** Anti-*malaria* activity of quinolones.

**Number**	a **pEC**_50 _**Exp.**	b **pEC**_50 _**Calc**	c **|REP|**
1	3.9208	3.86037	0.0157
2	1.1163	1.41986	0.2138
3	2.3556	4.94160	0.5233
4	4.8239	4.59390	0.0500
5	2.1421	2.19951	0.0261
6	2.5575	2.45931	0.0399
7	2.70996	3.33258	0.1868
8	2.0985	2.30913	0.0912
9	1.1101	1.36155	0.1846
10	0.2125	0.43881	0.5156
11	1.1391	1.11276	0.0236
12	1.21896	0.83808	0.4545
13	2.3615	2.06961	0.1410
14	2.0376	1.4404	0.4146
15	2.1469	1.56789	0.3693
16	1.1169	1.19209	0.0631
17	0.9931	1.19209	0.1669
18	1.2306	1.19209	0.0323
19	0.6108	0.6373	0.0415
20	0.6003	0.3849	0.5597
21	0.9991	1.23954	0.1939
22	0.3925	1.16511	0.6631
23	1.3233	1.48233	0.1073
24	1.8539	1.95589	0.0522
25	0.7878	1.27179	0.3805
26	2.5100	1.82599	0.3746
27	1.2660	1.14189	0.1087
28	1.8928	0.528	2.5848

a The experimentally activity (pEC_50_) in TM90-C2B.

b The calculated pEC_50_ using multi linear regression equation 1.

c The absolute value of Percent of the relative error of prediction.

**Table 4:** Pearson correlation coefficient matrix for the descriptors of quinolone used in the MLR activity equation 1.

**Correlations**							
		pEC_50_	PJI2	Mv	PCR	nBM	VAR
Pearson Correlation	pEC_50_	1.000	-0.514	-0.209	0.183	-0.226	-0.290
	PJI2	-0.514	1.000	-0.377	-0.135	0.140	0.305
	Mv	-0.209	-0.377	1.000	0.367	0.405	0.236
	PCR	0.183	-0.135	0.367	1.000	0.833	0.649
	nBM	-0.226	0.140	0.405	0.833	1.000	0.935
	VAR	-0.290	0.305	0.236	0.649	0.935	1.000
Sig. (1-tailed)	pEC_50_	.	0.003	0.143	0.176	0.123	0.067
	PJI2	0.003	.	0.024	0.247	0.239	0.057
	Mv	0.143	0.024	.	0.027	0.016	0.113
	PCR	0.176	0.247	0.027	.	0.000	0.000
	nBM	0.123	0.239	0.016	0.000	.	0.000
	VAR	0.067	0.057	0.113	0.000	0.000	.
N	pEC_50_	28	28	28	28	28	28
	PJI2	28	28	28	28	28	28
	Mv	28	28	28	28	28	28
	PCR	28	28	28	28	28	28
	nBM	28	28	28	28	28	28
	VAR	28	28	28	28	28	28

*QSTR equation*

Based on the procedure explained in the Materials and Methods section, using a stepwise multiple linear regression method, the following three-parametric equation 2 was derived for quinolones 1 to 28. 

Equation (2):

pEC_50_= (-7.35± 0.657) + (22.5 ± 1.46) MSD + (0.815 ± 0.085) MAXDP – (0.097 ± 0.014) X1sol

 n=18, F=81.14, R^2^=0.96, S=0.049, p<0.000, q2=0.812

 Equation 2 explains 96% of the variance in pEC_50_ data which describes the effect of MSD, MAXDP and X1sol indices on cytotoxicity. MSD and MAXDP are among topological descriptors and X1sol is among connectivity indices. The relative error prediction (REP) of the equation 2 is shown in Table 5.

**Figure 2:**
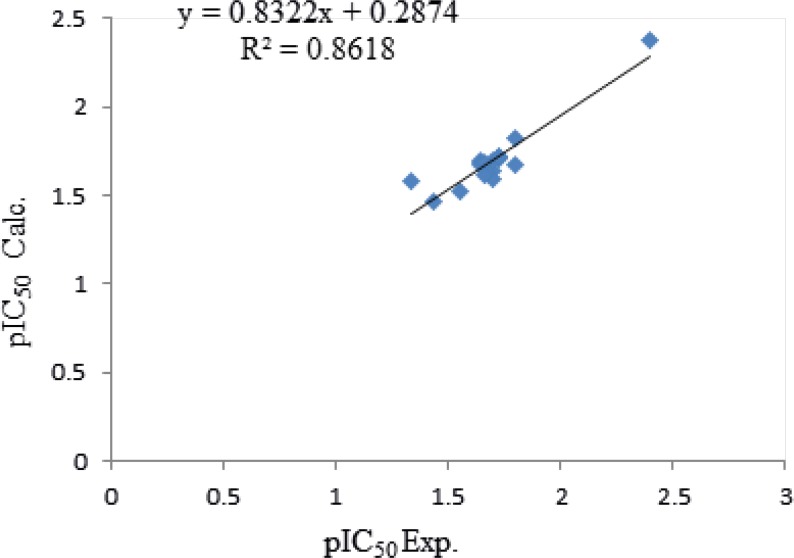
Plot of cross-validated calculated activity of Cytotoxicity obtained by QSTR equation 2.

 Equation 2 indicates that MSD and MAXDP demonstrate positive contribution and X1sol show a negative contribution towards the cytotoxicity which all descriptors have positive concept, so comparison of the coefficient of descriptors reveals that which cytotoxicity might be affected mainly by MSD. The calculated pEC_50_ using equation 2 is presented in Table 5 and the graphical representation of cross validated calculated activities and the experimental values using equation 2 are presented in Figure 2. The correlation coefficient matrix for the descriptors that were used in the MLR equation 2 is shown in Table 6. Based on this equation in order to have a new ligand with low cytotoxicity, some substitution with low amounts of MSD properties should be considered.

**Table 5:** Cytotoxicity of quinolone in term of pEC_50_.

**Number**	a **pEC**_50_ ** Exp.**	b **pEC**_50_ ** Calc.**	c **|REP|**
1	1.337242	1.729347	0.226736
2	1.431798	1.490277	0.039240
3	1.552842	1.741733	0.108450
4	3	1.736874	0.727241
5	1.721246	1.637286	0.051280
6	2.397940	2.237676	0.071621
7	1.638272	1.680007	0.024842
8	1.638272	1.675107	0.021989
9	1.638272	1.673392	0.020987
10	1.698970	1.645639	0.032407
11	1.698970	1.705242	0.003678
12	1.721246	1.812865	0.050538
13	1.795880	1.975504	0.090926
14	1.795880	1.811327	0.008528
15	1.698970	1.584743	0.072079
16	1.657577	1.640181	0.010606
17	1.721246	1.612183	0.067649
18	1.657577	1.5981	0.037217

a The experimentally cytotoxicity pEC_50 _in J774.

b The calculated pEC_50_ using multi linear regression equation 2.

c The absolute value of Percent of the relative error of prediction**.**

**Table 6:** Pearson correlation coefficient matrix for the descriptors of quinolones used in the MLR cytotoxicity equation 2.

**Correlations**					
		pEC_50_	MSD	MAXDP	X1sol
Pearson Correlation	pEC_50_	1.000	0.379	-0.068	-0.043
	MSD	0.379	1.000	-0.686	-0.484
	MAXDP	-0.068	-0.686	1.000	0.931
	X1sol	-0.043	-0.484	0.931	1.000
Sig. (1-tailed)	pEC_50_	.	0.060	0.394	0.432
	MSD	0.060	.	0.001	0.021
	MAXDP	0.394	0.001	.	0.000
	X1sol	0.432	0.021	0.000	.
N	pEC_50_	18	18	18	18
	MSD	18	18	18	18
	MAXDP	18	18	18	18
	X1sol	18	18	18	18

## Conclusion

Twenty eight analogs of quinolone with anti-*malaria* activities were subjected to QSAR and QSTR studies in order to design new ligands with an improved therapeutic index. Based on our QSAR and QSTR equations (Equations 1 and 2) PJI2, Mv, PCR, nBM, and VAR can effect on anti-*malaria *activity and MSD, MAXDP, and X1sol can effect on cytotoxicity. Our QSAR and QSTR calculations reveals in order to increase the therapeutic index of the series of anti-*malaria* compounds, some moieties with high amount of PCR and low amounts of Mv, PJI2, and MSD should be considered in the lead compounds.
